# Spectroscopic evidence of the effect of hydrogen peroxide excess on the coproheme decarboxylase from actinobacterial *Corynebacterium diphtheriae*

**DOI:** 10.1002/jrs.6326

**Published:** 2022-03-08

**Authors:** Federico Sebastiani, Chiara Niccoli, Hanna Michlits, Riccardo Risorti, Maurizio Becucci, Stefan Hofbauer, Giulietta Smulevich

**Affiliations:** ^1^ Dipartimento di Chimica “Ugo Schiff” DICUS Università di Firenze Sesto Fiorentino Italy; ^2^ Department of Chemistry, Institute of Biochemistry University of Natural Resources and Life Sciences, Vienna Vienna Austria; ^3^ INSTM Research Unit of Firenze Sesto Fiorentino Italy

**Keywords:** chlorin, heme *b*, oxidative damage, porphyrin hydroxylation, resonance Raman

## Abstract

The actinobacterial coproheme decarboxylase from *Corynebacterium diphtheriae* catalyzes the final reaction to generate heme *b* via the “coproporphyrin‐dependent” heme biosynthesis pathway in the presence of hydrogen peroxide. The enzyme has a high reactivity toward H_2_O_2_ used for the catalytic reaction and in the presence of an excess of H_2_O_2_ new species are generated. Resonance Raman data, together with electronic absorption spectroscopy and mass spectrometry, indicate that an excess of hydrogen peroxide for both the substrate (coproheme) and product (heme *b*) complexes of this enzyme causes a porphyrin hydroxylation of ring C or D, which is compatible with the formation of an iron chlorin‐type heme *d* species. A similar effect has been previously observed for other heme‐containing proteins, but this is the first time that a similar mechanism is reported for a coproheme enzyme. The hydroxylation determines a symmetry lowering of the porphyrin macrocycle, which causes the activation of A_2g_ modes upon Soret excitation with a significant change in their polarization ratios, the enhancement and splitting into two components of many E_u_ bands, and an intensity decrease of the non‐totally symmetric modes B_1g_, which become polarized. This latter effect is clearly observed for the isolated ν_10_ mode upon either Soret or Q‐band excitations. The distal His118 is shown to be an absolute requirement for the conversion to heme *d*. This residue also plays an important role in the oxidative decarboxylation, because it acts as a base for deprotonation and subsequent heterolytic cleavage of hydrogen peroxide.

## Graphical abstract

Coproheme and heme *b* complexes of coproheme decarboxylase from *Corynebacterium diphtheriae* in the presence of an excess of H_2_O_2_ generate an iron chlorin‐type heme *d* species. The heme *d* formation determines a symmetry lowering of the porphyrin macrocycle with activation of A_2g_ modes upon Soret excitation, splitting and enhancement of E_u_ bands, and an intensity decrease of the non‐totally symmetric modes B_1g_, which become polarized. The distal His118 is an absolute requirement for the conversion to heme *d* in the presence of excess H_2_O_2_.

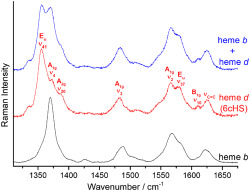

**Abbreviations**
5c: five‐coordinated
6c: six‐coordinated
*Cd*ChdC: coproheme decarboxylase from *Corynebacterium diphtheriae*
ChdC: coproheme decarboxylase
eq.: equivalents
HS: high‐spin
IR: infrared
LC–MS: liquid chromatography–mass spectrometry
MMD: monovinyl monopropionyl deuteroheme
MS: mass spectrometry
NMR: nuclear magnetic resonance
QS: quantum mixed‐spin
RR: resonance Raman
WT: wild‐type

## 1 INTRODUCTION

**FIGURE 1:**
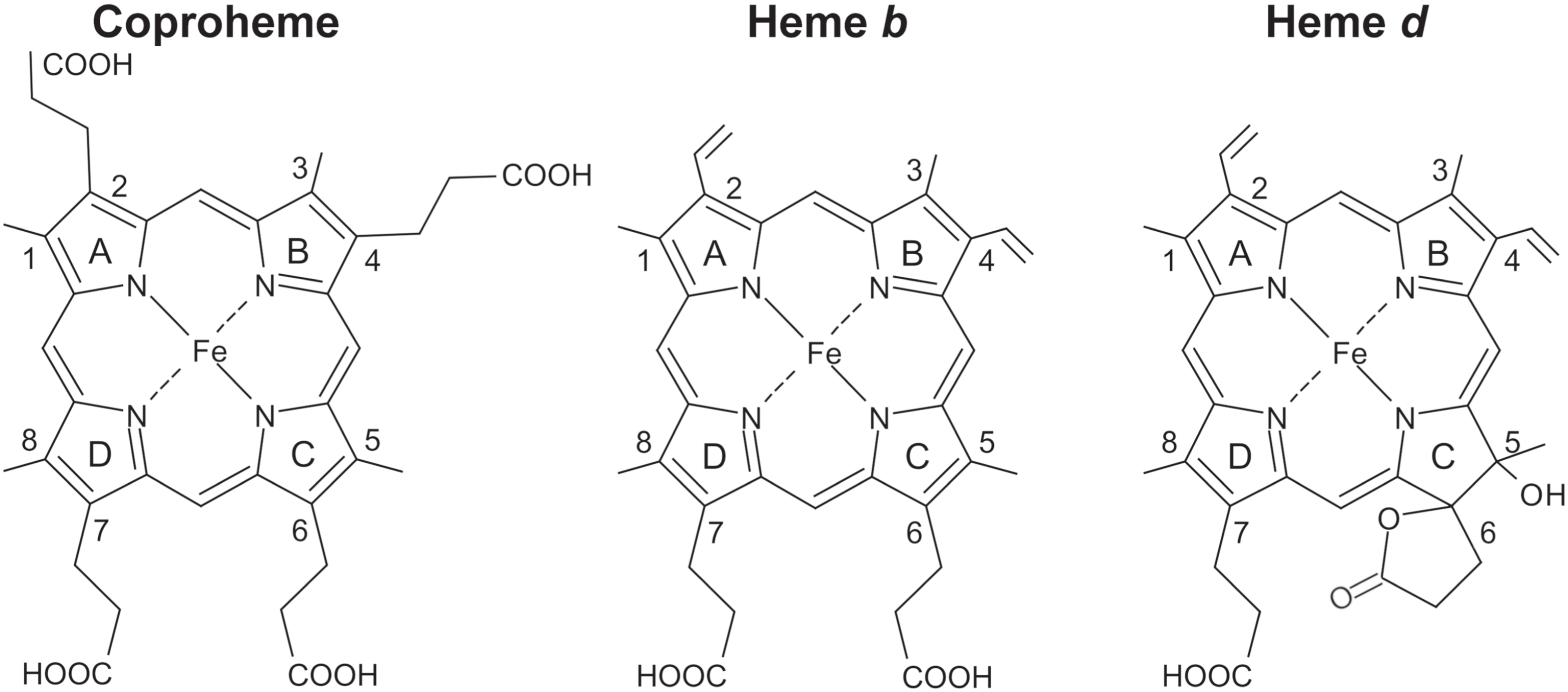
Structures of coproheme, heme *b*, and heme *d*. Numbering of the pyrrole rings and carbon atoms follows the Fischer nomenclature. In isolated heme *d*, the propionate at position 6 or 7 and hydroxyl side groups can form a lactone on ring C or D

Many heme proteins use hydrogen peroxide to perform their enzymatic activity, namely, peroxidases, catalases, and decarboxylases.^[^ ^1^, ^2^ ^]^ For coproheme decarboxylases (ChdCs), hydrogen peroxide has been described as the most probable physiological oxidant to start the decarboxylation reaction, which represents the last step of heme biosynthesis in the so‐called “coproporphyrin‐dependent” pathway of Gram‐positive bacteria.^[^ ^3^, ^4^, ^5^, ^6^ ^]^ However, the addition of hydrogen peroxide can promote oxidative damage and subsequent potential cytotoxicity^[^ ^7^, ^8^, ^9^ ^]^ and, at a molecular level, can also alter the heme group. In fact, many hemeproteins (e.g., hemoglobin, myoglobin, and cytochrome c) in the presence of exogenous hydrogen peroxide rapidly form covalently cross‐linked dimers and polymers detectable by detergent gel electrophoresis.^[^ ^10^, ^11^, ^12^ ^]^ Moreover, an iron chlorin‐type heme *d* product has been reported for metmyoglobin,^[^ ^13^ ^]^ for hemoglobin both in vitro and in vivo,^[^ ^14^ ^]^ for cytochrome *bd*,^[^ ^15^ ^]^ and for *Penicillium vitale* catalase and *Escherichia coli* catalase hydroperoxidase II (HPII).^[^ ^16^, ^17^, ^18^, ^19^ ^]^ Heme *d* (Figure 1) is a derivative of heme *b*, which carries an additional hydroxyl group and a γ‐spirolactone originating from the propionate side chain on a pyrrole ring.^[^ ^20^ ^]^ Therefore, due to the modification of a ring, heme *d* lacks one of the double bonds of the porphyrin system and, therefore, belongs to the tetrapyrrole subtype of chlorins.

The iron chlorin product obtained by the peroxide‐mediated alteration of the ferric myoglobin heme has been characterized by different spectroscopic methods including UV–vis, mass spectrometry (MS), infrared (IR), and nuclear magnetic resonance (NMR) spectroscopies.^[^ ^20^ ^]^ MS evidenced the presence of two products, one with a molecular ion at m/z 632.5 corresponding to the addition of one oxygen to heme *b* and the other with a molecular ion at m/z 650.2 corresponding to the addition of two oxygens and two protons to heme *b*.

Recently, it has been shown that coproheme decarboxylases from actinobacterial *Corynebacterium diphtheriae* (*Cd*ChdCs) have a remarkably high reactivity toward H_2_O_2_ used for the catalytic reaction. In monoderm bacteria, ChdCs catalyze the final reaction to generate heme *b* via the “coproporphyrin‐dependent” heme biosynthesis pathway in presence of hydrogen peroxide.^[^ ^3^, ^21^, ^22^ ^]^ Coproheme (iron coproporphyrin III) differs from heme *b* for the presence of four propionates at positions 2, 4, 6, and 7, instead of two vinyls and two propionates (Figure 1). ChdC promotes heme *b* formation from coproheme by stepwise decarboxylation of the propionate groups at positions 2 and 4 of the pyrrole rings A and B into vinyls. In addition, it has been reported that the coproheme complex of the catalytically inactive *Cd*ChdC Y135A variant titrated with H_2_O_2_ does not accumulate heme *b* but a new species, whose chemical and electronic structures remain unknown.^[^ ^23^ ^]^

The wild‐type (WT) heme *b*‐containing *Cd*ChdC protein, upon addition of an excess of exogenous hydrogen peroxide, gives rise also to a new species.^[^ ^24^ ^]^ Therefore, in this paper, we have investigated further the formation of this new species formed in the excess of hydrogen peroxide in both the *Cd*ChdC Y135A variant coproheme and the WT heme *b* complexes by means of MS together with UV–vis electronic absorption and resonance Raman (RR) spectroscopies.

We will show that RR is particularly powerful and sensitive to study these processes because the changes observed in intensity and in the polarization ratios of vibrational modes can be connected to the change of the symmetry of the porphyrin ring that lowers from the pseudosymmetry D_4h_ to a C_2_′ symmetry group, with the increase of a substantial distortion. Our results clearly demonstrate that both *Cd*ChdC coproheme and heme *b* complexes, in the presence of an excess of hydrogen peroxide, undergo the formation of an iron chlorin‐type heme *d* species, similar to ferric myoglobin, hemoglobin, and catalases. Iron chlorin complexes have been characterized by RR spectroscopy by Andersson and coworkers, as models for the prosthetic groups of green heme proteins.^[^ ^25^ ^]^ Comparing the iron chlorin RR spectra to those of the iron (III)–protoporphyrin IX (heme *b*) complex, they found that the spin states of the iron (III)–chlorin complexes give rise to core size marker bands in the high‐frequency region analogous to those of the iron (III)–porphyrin complexes. Thus, the knowledge developed on spin and oxidation states marker bands of porphyrins could be transferred to chlorins. The frequencies of the so‐called “core size marker bands” allowed the heme/chlorins oxidation, coordination, and spin states to be identified. However, they found major differences in the relative intensity and in the depolarization ratios of these bands. Therefore, in the present work, we characterize the new species obtained for *Cd*ChdC in presence of an excess of hydrogen peroxide, in their ferric forms, by RR excitation in the Soret and in the Q‐band regions, using non‐polarized and polarized light. The results are then compared with those obtained for the corresponding ferrous forms.

## 2 MATERIALS AND METHODS

### 2.1 Expression and purification of WT *Cd*ChdC and variants

Cloning, site‐directed mutagenesis, and expression of the *Cd*ChdC gene (DIP1394) and its variants Y135A and Y135A/H118F were performed as described in detail previously.^[^ ^23^, ^24^ ^]^

### 2.2 Sample preparation

Ferric coproheme–*Cd*ChdC samples of WT, Y135A, and Y135A/H118F variants were prepared by adding free coproheme solution (0.5 M NaOH) to apo‐*Cd*ChdC diluted in 50 mM phosphate buffer, pH 7.0 in a 2:1 apoprotein:coproheme ratio. Coproheme–*Cd*ChdC samples were titrated by adding small aliquots of a 1 mM H_2_O_2_ solution in 50 mM phosphate buffer, pH 7.0. The WT coproheme complex was titrated to 1.75 equivalents (eq.) H_2_O_2_ to obtain the WT heme *b* complex, instead of the 2 eq. H_2_O_2_ that has been reported previously,^[^ ^24^ ^]^ because at 2 eq. of hydrogen peroxide, a minor amount of oxidized heme *b* already forms (see below). In detail, the Y135A coproheme complex was titrated to an excess of 2 eq. of H_2_O_2_, whereas the WT heme *b* complex was further titrated to an excess of 1.75 eq. of H_2_O_2_, to obtain the oxidized forms. Above such amounts of hydrogen peroxide, denaturation of the proteins started to occur.

The ferrous and CO‐adduct samples were prepared by addition of a freshly prepared sodium dithionite (20 mg ml^−1^) solution to the ferric forms previously degassed with nitrogen or CO (Rivoira, Milan, Italy), respectively. The ferric, ferrous, and CO‐adduct sample concentrations were in the range 30–60 μM.

### 2.3 UV–vis electronic absorption

UV–vis electronic absorption spectra were recorded using a 5 mm NMR tube or a 1 mm cuvette (600 nm min^−1^ scan rate) at 25°C by means of a Cary 60 spectrophotometer (Agilent Technologies, Santa Clara, CA) with a resolution of 1.5 nm. All the spectra were normalized to the intensity of the Soret band and the 450–700 nm region in all the absorption spectra has been further magnified by a factor from 5‐ to 15‐fold, depending on the sample.

### 2.4 Resonance Raman

RR spectra were obtained at room temperature using a slowly rotating 5 mm NMR tube by excitation with the 406.7 and 413.1 nm lines of a Kr^+^ laser (Innova300 C; Coherent, Santa Clara, CA), the 441.6 nm line of a He–Cd laser (IK4121R‐G; Kimmon Koha, Tokyo, Japan), and the 532 nm line of a diode laser (Cobolt Samba 300). Back‐scattered light was collected and focused into a triple spectrometer (Acton Research, Acton, MA), consisting of two SpectraPro 2300i instruments working in the subtractive mode and a SpectraPro 2500i instrument in the final stage with a grating of 3600 or 1800 grooves mm^−1^, and equipped with a liquid nitrogen‐cooled charge‐coupled device detector. A spectral resolution of 1.2 and 4 cm^−1^ was calculated on the basis of the optical properties of the spectrometer for the 3600 and 1800 grooves mm^−1^ gratings, respectively. The RR spectra were calibrated with indene and carbon tetrachloride as standards to an accuracy of 1 cm^−1^ for intense isolated bands. Spectra in polarized light were obtained by inserting a polaroid analyzer between the sample and the entrance slit of the monochromator. The depolarization ratios of the bands at 314 (*ρ* = 0.75) and 460 cm^−1^ (*ρ* ≈ 0) of CCl_4_^[^ ^26^ ^]^ were measured to check the reliability of the measurements. To avoid sample denaturation, a stream of cooled nitrogen gas was directed toward the rotating sample tube (15°C).

All RR measurements were repeated several times under the same conditions to improve the signal‐to‐noise ratio, and the spectra were summed only if no spectral differences were noted. Table S1 summarizes the integration time and the number of averaged spectra reported in the figures. The electronic absorption spectra were measured both before and after RR measurements to check that no degradation of the sample occurred under the experimental conditions used.

All spectra were baseline corrected. In particular, for the spectra obtained with the 532 nm excitation, the blank spectrum of the buffer was subtracted (in both polarized and non‐polarized light) from those of the samples to obtain a reliable evaluation of the relative intensities of the vibrational bands.

The RR spectra upon 406.7, 413.1, and 441.8 nm excitation were normalized to the intensity of the ν_4_ band for the high wavenumber region and to the ν_7_ band in the low wavenumber region, respectively, whereas upon 532 nm excitation, the RR spectra were normalized to the most intense band of the spectrum at about 1560 cm^−1^ (ν_11_ or ν_19_ depending on the sample).

To avoid photoreduction, a 5.5 mW laser power at the sample was used for the ferric coproheme complexes of the WT and the variants, whereas a 6.5 mW laser power at the sample was employed for the ferric complexes upon titration with hydrogen peroxide for the WT and the variants. Using these conditions, no change in the spectra have been observed upon repeated measurement sessions. The power on the ferrous samples was 3.5 and 5.5 mW for coproheme complexes and H_2_O_2_‐titrated complexes, respectively.

### 2.5 Mass spectrometry

Titrations to determine the reaction of hydrogen peroxide with *Cd*ChdC WT and variants were performed and monitored via MS as previously described in detail.^[^ ^4^ ^]^ The liquid chromatography–mass spectrometry (LC–MS) method was optimized in a way to detect small molecules (e.g., coproheme, oxidized coproheme, monovinyl monopropionyl deuteroheme [MMD], heme *b*, and oxidized heme *b*) in the range from 500 to 1000 Da, as well as the entire protein (approximately 27–28 kDa), to detect protein modifications, such as heme cross‐linking, in one run.

### 2.6 Pre‐steady state kinetics of coproheme oxidation

The spectral transitions, which occur during the oxidation of coproheme, were monitored using stopped‐flow apparatus equipped with a photodiode array detector (SX‐18MV, Applied Photophysics, Leatherhead, UK). The pathlength and volume of the optical quartz cell were 10 mm and 20 μl, respectively. The dead time of the system is 1 ms, which was also the timepoint for the first obtained spectrum. All measurements were performed in 50 mM phosphate buffer, pH 7.0, at room temperature. In a typical representative experiment, 2 μM of *Cd*ChdC Y135A coproheme complex were mixed in the cell with 10 μM hydrogen peroxide. The rate constant for this reaction was determined using a stopped‐flow apparatus equipped with a monochromator and a photomultiplier (Pi‐star, Applied Photophysics, Leatherhead, UK). This allows to reliably follow changes in UV–vis absorption at a single wavelength (392 or 580 nm) over time, thereby eliminating potential photobleaching. Typically, the enzyme concentration was 2 μM for the *Cd*ChdC Y135A coproheme complex and the concentration of hydrogen peroxide was varied step‐wise from 5 to 50 μM. For the determination of the oxidation reaction of coproheme, the wavelength 580 nm was chosen as the single wavelength and a minimum of three repetitions were performed for each hydrogen peroxide concentration. *k*_obs_ values for each substrate/oxidant concentration were determined by fitting the respective time traces with a double‐exponential curve. The first exponential phase represents Compound I formation and the second oxidation of coproheme. The *k*_obs_ values were plotted against the respective hydrogen peroxide concentration to obtain the apparent rate constant of the formation of oxidized coproheme.

## 3 RESULTS

**FIGURE 2:**
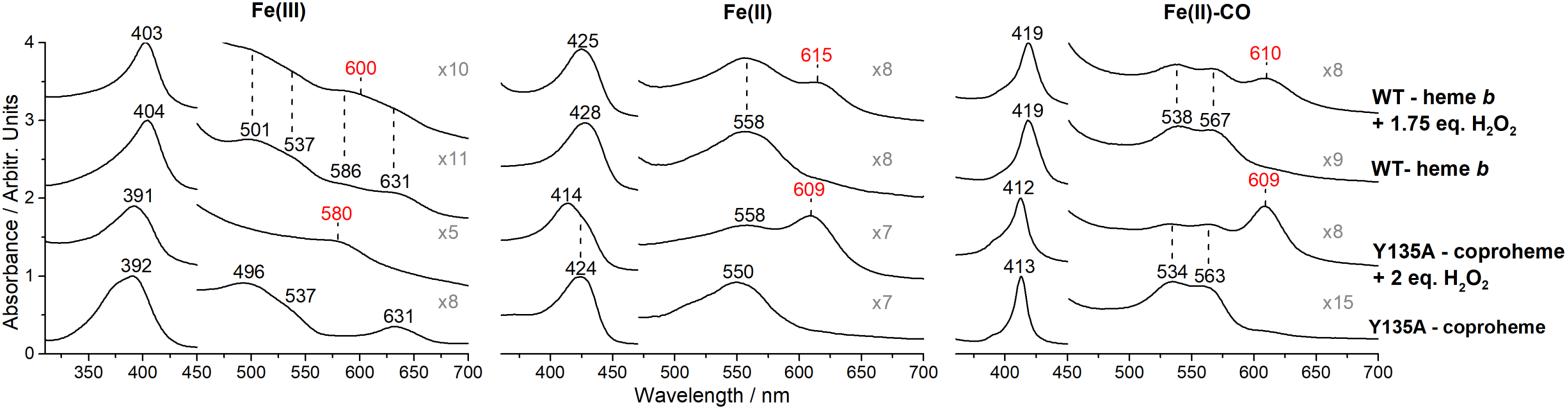
Comparison of the UV–vis electronic absorption spectra of the ferric (left), ferrous (middle), and CO adduct (right) of the *Cd*ChdC WT heme *b* and its Y135A variant coproheme complexes treated with an excess of hydrogen peroxide. The new band formed in the presence of an excess of hydrogen peroxide is marked with red numbers. All the spectra have been normalized to the intensity of the Soret band, and the 450–700 nm region has been magnified as indicated [Colour figure can be viewed at wileyonlinelibrary.com]

The hydrogen peroxide‐dependent oxidative radical decarboxylation by ChdC generates heme *b* from ferric coproheme. This oxidative decarboxylation has been fully characterized by UV–vis and RR spectroscopies in monoderm bacteria.^[^ ^3^, ^24^, ^27^, ^28^ ^]^ After cleavage of the propionate at position 2, the transiently formed three‐propionate intermediate, MMD undergoes a 90° rotation within the active site before the decarboxylation of the propionate at position 4. This reorientation is essential for bringing the propionate at position 4 in close proximity to the catalytic residue (Y135) and, consequently, for its decarboxylation by the Y135 radical to lead to the formation of heme *b*.^[^ ^22^, ^24^ ^]^ The electronic absorption spectra of the coproheme complexes of the ferric WT *Cd*ChdC and its variant Y135A are very similar and characteristic of a five‐coordinated (5c) species. In fact, RR spectra identified a 5c high spin (HS) in equilibrium with a 5c quantum mixed‐spin (QS) state (Figure 2, left, Figure S1, and reference^[^ ^24^ ^]^). Upon titration of the WT coproheme with hydrogen peroxide, the WT heme *b* complex is obtained, with a UV–vis absorption spectrum typical of an HS species, red‐shifted as compared with its coproheme counterpart due to the vinyl double bond conjugation (Figure 1, Figure 2, left, and Figure S1). The corresponding RR spectrum of the WT heme *b* complex identified the presence of a mixture of 5cHS and 6cHS species.^[^ ^24^ ^]^

To understand the effect of an excess of hydrogen peroxide and its implications, we investigated the *Cd*ChdC complexes of both the substrate (coproheme) and the product of the decarboxylation reaction (heme *b*).

As the Y135A *Cd*ChdC variant is catalytically inactive, the addition of hydrogen peroxide does not produce a heme *b* complex, but other species, as suggested by mass spectra.^[^ ^23^ ^]^ The presence of a new species is presently confirmed by further spectral changes. The effect of an excess of hydrogen peroxide induces a decrease of absorbance of the overall UV–vis absorption spectra of both *Cd*ChdC Y135A variant coproheme and WT heme *b* complexes (about 20%) together with the appearance of a new band at 580 nm (Y135A coproheme complex) and 600 nm (heme *b* complex) in the visible region (Figure 2, left). The comparison of the UV–vis absorption spectra in Figure 2 clearly indicates that upon addition of an excess of H_2_O_2_, not only the ferric forms (left) but also the ferrous (middle) and CO adducts (right) of both the WT heme *b* and Y135A variant coproheme complexes are characterized by the appearance of a new band in the visible region. The bands of the heme *b* complexes resemble those found in the corresponding iron chlorin‐type heme *d*, which have maxima at about 602 nm (ferric), 613 nm (ferrous), and 614 nm (CO adduct).^[^ ^29^, ^30^ ^]^ The corresponding new bands of the coproheme complexes are at similar, although blue‐shifted, wavelengths as compared with the heme *b* counterparts. Assuming that an analogous reaction takes place in both systems, the lack of the double bond conjugation in the coproheme complexes, due the absence of the vinyl groups at positions 2 and 4, possibly leads to the observed blue‐shift.

**FIGURE 3:**
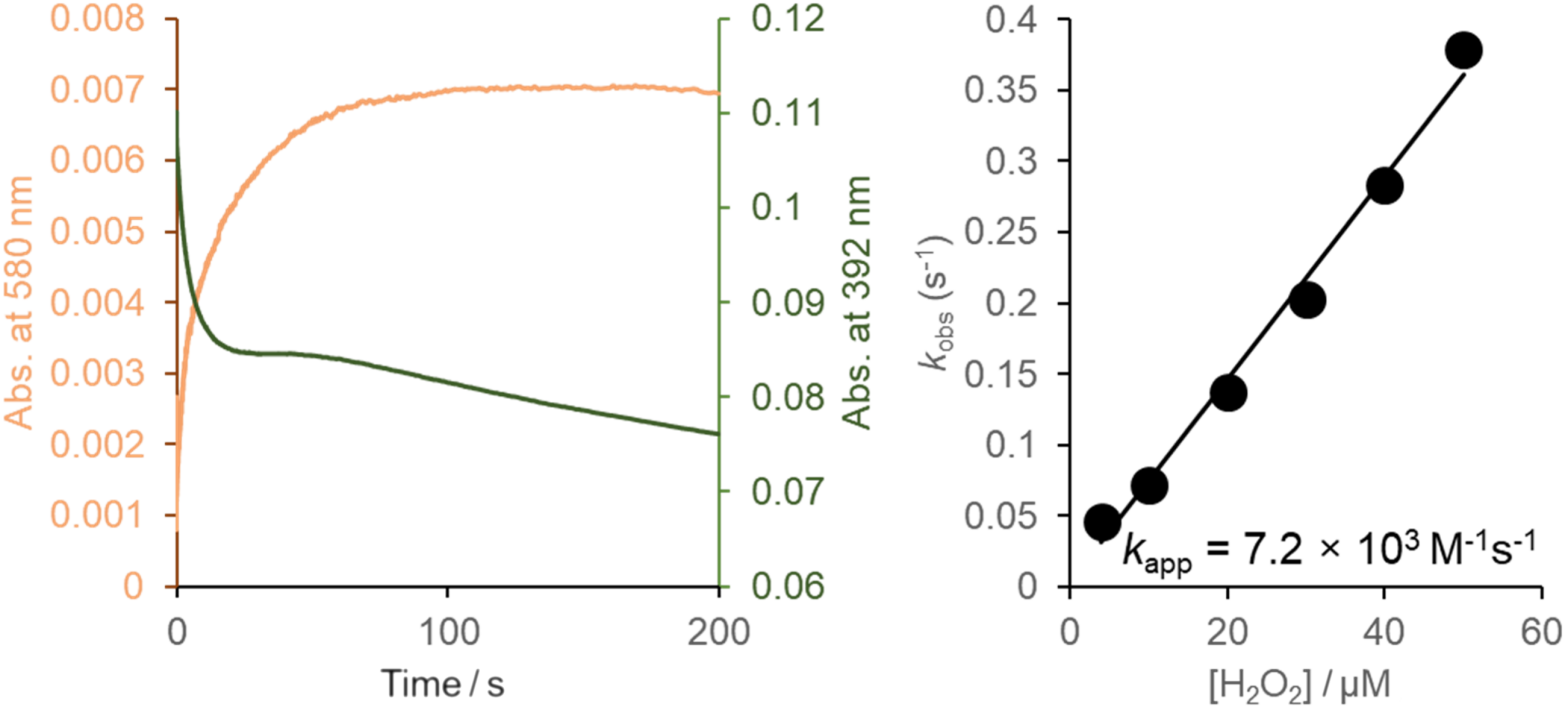
Hydrogen peroxide triggered oxidation of the *Cd*ChdC Y135A variant coproheme complex: (left) Representative time traces at 580 and 392 nm of 2 μM Y135A coproheme complex upon mixing with 10 μM hydrogen peroxide in 50 mM phosphate buffer, pH 7.0, as followed by stopped‐flow spectroscopy. (right) Linear dependence of *k*_obs_ values versus hydrogen peroxide concentration [Colour figure can be viewed at wileyonlinelibrary.com]

Furthermore, stopped‐flow spectroscopy allowed us to follow the spectral transitions of the *Cd*ChdC Y135A coproheme complex upon addition of hydrogen peroxide (Figure 3 and reference^[^ ^21^ ^]^). The overall changes of the spectrum include an absorbance loss of the Soret band (392 nm) and the appearance of a new band at 580 nm due to the oxidation of coproheme. The time traces at 580 nm allowed us to determine the rate of the reaction of coproheme (Figure 3, left). Following the reaction at 392 nm, we were able to track Compound I formation.^[^ ^23^ ^]^ The rate of formation of the oxidized coproheme species, as determined by stopped‐flow UV–vis spectroscopy, yields a *k*_app_ of 7.2 × 10^3^ M^−1^ s^−1^ (Figure 3, right).

**FIGURE 4:**
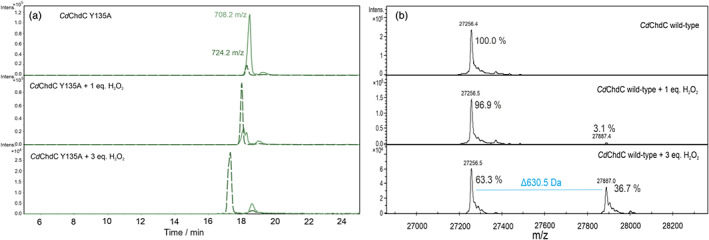
(a) Chromatograms of the *Cd*ChdC Y135A variant untreated and treated with hydrogen peroxide. Elution of free coproheme in its native state (708.2 Da) is shown as a green solid line, and free oxidized coproheme as a dark green dashed line (724.2 Da). (b) Mass spectrometric data of whole protein measurements of *Cd*ChdC wild‐type untreated and treated with hydrogen peroxide [Colour figure can be viewed at wileyonlinelibrary.com]

We have further characterized the oxidized forms by MS (Figure 4).

Figure 4 shows both the chromatograms for the masses of coproheme and oxidized coproheme complexes obtained by LC–MS for the *Cd*ChdC Y135A variant (a) and the mass spectrometric data obtained for the WT (b) complexes upon addition of hydrogen peroxide. The MS analysis of the coproheme complex of the *Cd*ChdC Y135A variant upon increasing the H_2_O_2_ amount shows that no heme *b* is present, but instead a molecular ion at m/z 724.2 is formed at the expense of the coproheme (708.2 Da) (Figure 4a). The difference corresponds to the addition of an oxygen to the coproheme. Similarly, the presence of excess hydrogen peroxide for the heme *b* complex of the *Cd*ChdC WT leads to the formation of a new species, which can only be detected in whole protein measurements as the product is cross‐linked to the protein (Figure 4b, bottom spectrum). The detected subunit mass of the ChdC cross‐linked with a heme species is at m/z ion at 27887.0 Da, compared with that of the heme‐free ChdC of 27256.5 Da (a difference of 630.5 Da). This mass difference again corresponds to the addition of an oxygen to the heme *b*. The expected mass of free oxidized heme in solution is 632.2 Da, which is not found in the covalently linked form as the mass of one or two protons, depending on how many ester bonds between negatively charged residues with heme methyl groups are established, has to be subtracted.

We will start the analysis of the RR spectra from the wavenumber region between 1300 and 1700 cm^−1^, where the core size marker bands are localized. The core size marker bands, intensified by excitation in the Soret or Q bands, show different polarization ratios according to their A_1g_, B_1g_, A_2g_, and B_2g_ irreducible representations under the D_4h_ pseudosymmetry of the heme group.^[^ ^31^ ^]^ The full vibrational assignment of the spectra is based on the detailed analysis of the porphyrin modes, as obtained by isotopic labeling studies and systematic vibrational analysis of model nickel porphyrins with different peripheral substituents supported by ab initio computations.^[^ ^32^, ^33^, ^34^, ^35^, ^36^, ^37^ ^]^ The classical heme *b* proteins and model compounds show an enhancement of the polarized A_1g_ by Soret excitation (Franck–Condon scattering), whereas when in resonance with the Q bands, the non‐totally symmetric depolarized B_1g_ modes and the anomalous polarized A_2g_ modes are enhanced (Herzberg–Teller scattering). In addition, the depolarized B_1g_ modes give rise to weak bands upon Soret excitation as a consequence of an enhancement attributed to the Jahn–Teller effect.^[^ ^38^ ^]^ The two vinyl substituents are characterized by the polarized ν(C═C) stretching vibrations observed around 1620–1630 cm^−1^, which are expected to be intensified only by Soret excitation.^[^ ^31^ ^]^ Moreover, the vinyl groups are known to induce Raman activation of the E_u_ modes, which are usually only IR active.^[^ ^39^, ^40^ ^]^

**FIGURE 5:**
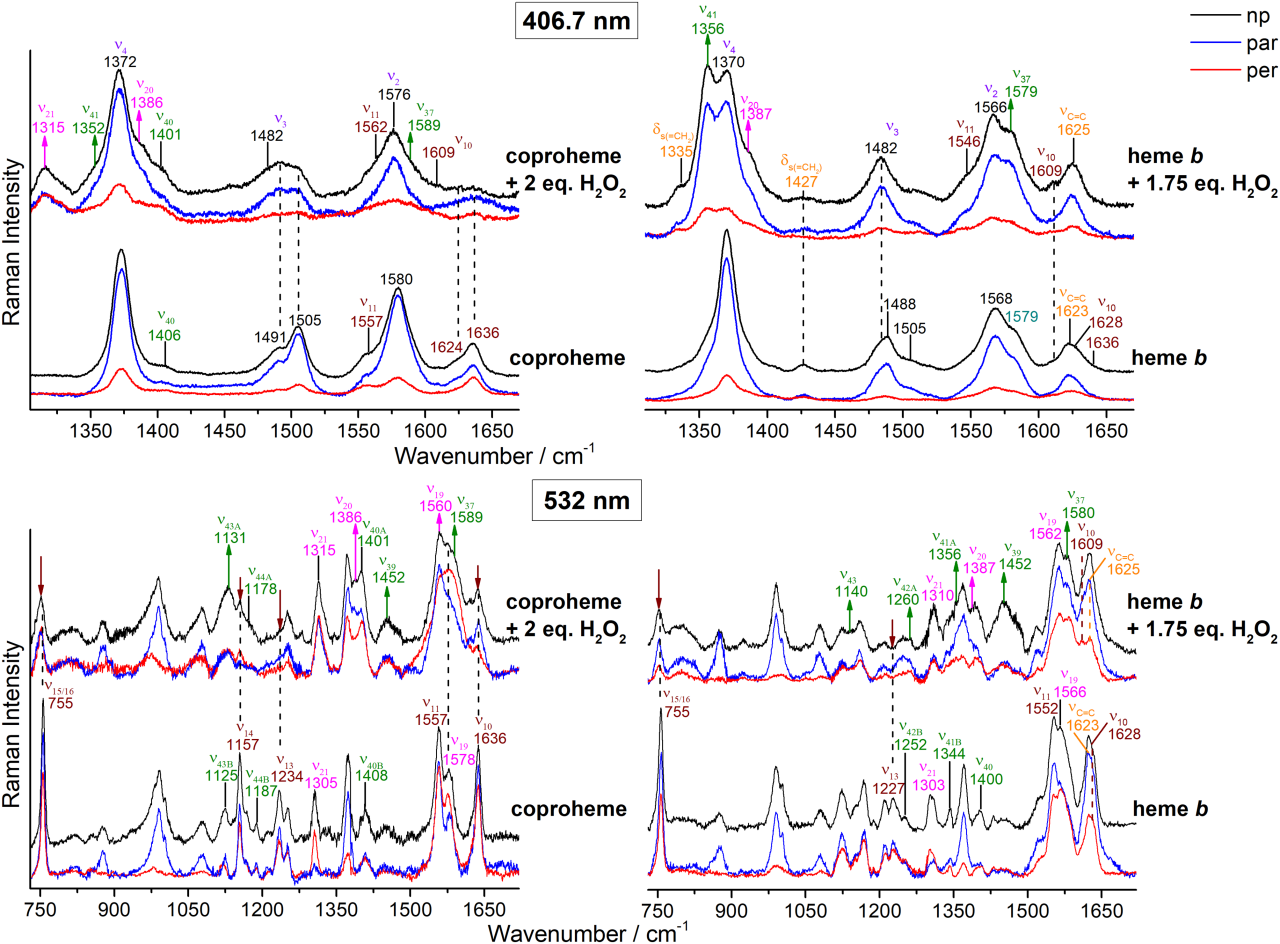
Resonance Raman spectra of ferric *Cd*ChdC coproheme (left) and heme *b* (right) complexes upon titration with H_2_O_2_, obtained with the 406.7 nm (top) and 532.0 nm (bottom) laser excitations. The spectra taken in polarized light are also reported in blue (parallel) and red (perpendicular). The vinyl stretching modes are reported in orange and the different irreducible representations of the vibrational modes are outlined with the following colors: A_1g_ (purple), A_2g_ (magenta), B_1g_ (maroon), and E_u_ (green). Arrows indicate the bands whose intensities increase or decrease upon addition of an excess of H_2_O_2_ [Colour figure can be viewed at wileyonlinelibrary.com]

**FIGURE 6:**
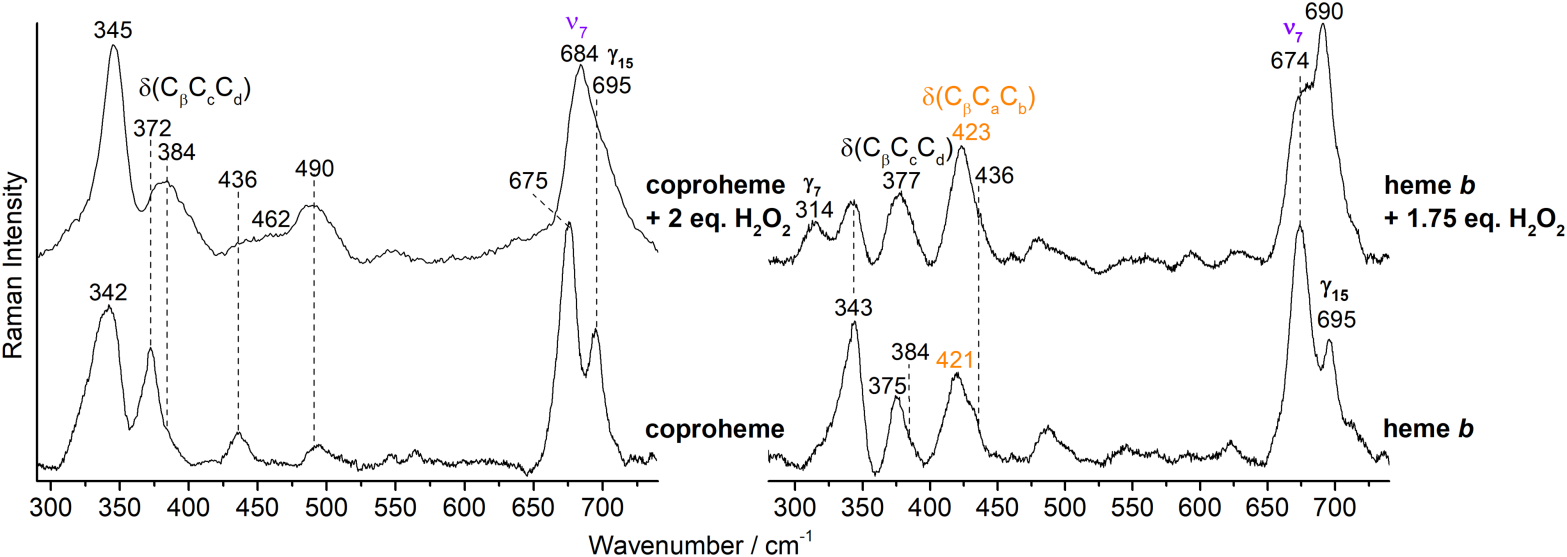
Resonance Raman spectra of ferric *Cd*ChdC coproheme (left) and heme *b* (right) complexes upon titration with H_2_O_2_ in the low wavenumber region obtained with the 406.7 nm laser excitation. The A_1g_ modes are reported in purple and the vinyl bending modes in orange [Colour figure can be viewed at wileyonlinelibrary.com]

Hereafter, we will indicate as coproheme, the coproheme complex of the *Cd*ChdC Y135A variant and as heme *b*, the heme *b* complex of the *Cd*ChdC WT, obtained as reported in Section 2. The alteration of the porphyrin macrocycle induced by an excess of H_2_O_2_ causes common changes in the spectra of both the coproheme and heme *b* complexes. In general, the spectra reveal an increase in the number of vibrational features, a variation of the relative intensities for some bands together with a change of their polarization ratios. In the high wavenumber region, the RR spectra obtained with Soret excitation (Figure 5, top spectra) clearly indicate that the excess hydrogen peroxide causes the formation of a new 6cHS species (ν_3_ and ν_10_ at 1482 and 1609 cm^−1^, respectively), whereas the contribution from the 5cHS and 5cQS populations are still evident. This means that three species coexist for the coproheme complexes: 5cHS, 5cQS, and 6cHS. Similarly, for the heme *b* complexes, there is a mixture of a predominant 6cHS together with a minor 5cHS species. The full assignment of the bands is reported in Figures 5 and 6; in the text, we will discuss only the most prominent effects for the sake of clarity.

The IR‐active E_u_ modes (labeled in green in Figure 5) increase in intensity upon addition of an excess of H_2_O_2_. This is particularly evident in the heme *b*‐complex spectra, where among the various intensified E_u_ modes, the ν_41_ at 1356 cm^−1^ gives rise to a polarized band, which becomes the strongest band of the overall spectrum, even more intense than the ν_4_ mode. An intensity increase is also observed for the A_2g_ bands (in magenta in Figure 5), which become active upon Soret excitation gaining substantial intensity. The ν_20_ at 1386–7 cm^−1^, which appears to be depolarized, is also strongly intensified. Usually, the A_2g_ bands (anomalously polarized) are intensified only upon Q‐band excitation, unless the porphyrin macrocycle undergoes a severe distortion as observed, for example, in the saddle shape‐distorted cytochrome c^[^ ^41^ ^]^ or human myeloperoxidase.^[^ ^42^ ^]^ Moreover, the RR spectra taken in polarized light suggest that the ν_10_ mode, which under the D_4h_ pseudosymmetry of the heme group is a depolarized B_1g_ mode, becomes polarized in the coproheme‐complex spectra in the presence of excess hydrogen peroxide (Figure 5, top left panel). RR excitation in the Q‐band region (Figure 5, bottom) confirms all these effects observed when an excess of hydrogen peroxide is present: Many E_u_ modes become RR active, and some of them split into doublets; the A_2g_ modes gain intensity and, in many cases, do not maintain their anomalous polarization. This is clearly observed for the more intense and isolated A_2g_ bands, the ν_19_ and ν_20_, which become depolarized. The B_1g_ modes are less intense, and the isolated B_1g_ ν_10_ mode of the coproheme complex is clearly polarized. The polarization of the corresponding mode of the heme *b* complex in presence of an excess of H_2_O_2_ is more difficult to evaluate. In fact, the ν_10_ mode overlaps with the ν(C═C) stretching modes, which remain active at both excitation wavelengths. It is worth noting that the ν(C═C) stretching modes at 1623 cm^−1^ in the heme *b*‐complex spectra upshift by 2 wavenumbers, when an excess of hydrogen peroxide is present.

In the low wavenumber region, between 300 and 700 cm^−1^, the in‐plane and out‐of‐plane bending vibrations of the porphyrin can be identified together with the bending modes of the heme peripheral groups, that is, the δ(C_β_C_c_C_d_) for the propionates and the δ(C_β_C_a_C_b_) for the vinyls. In addition, only few totally symmetric (A_1g_) modes are present, namely, the ν_8_ (343–345 cm^−1^) and ν_7_ (around 675 cm^−1^). Comparison of the results obtained for the coproheme and heme *b* complexes in the low wavenumber region (Figure 6) clearly shows that the spectra differ due to the presence of the δ(C_β_C_a_C_b_) vinyl bending modes in the heme *b* case (Figure 6, right, indicated in orange). These bending modes undergo a slight wavenumber upshift upon addition of 1.75‐fold excess of hydrogen peroxide, in agreement with the changes observed for the corresponding stretching modes in the high wavenumber region. Moreover, a small rearrangement of the propionate peripherical substituents is also suggested by the change of their δ(C_β_C_c_C_d_) bending mode frequencies in both the coproheme‐ and heme *b*‐complex spectra upon addition of an excess peroxide. However, the most dramatic change in this region is the upshift of the totally symmetric mode (ν_7_) assigned to δ(pyr_deform_)_sym_.^[^ ^34^ ^]^

**FIGURE 7:**
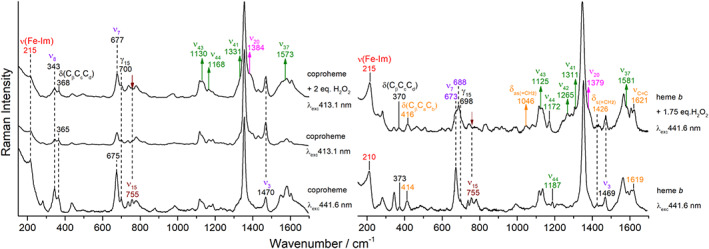
Resonance Raman spectra of ferrous CdChdC coproheme (left) and heme *b* (right) complexes upon titration with H_2_O_2_ at different excitation wavelengths (*λ*_exc_). The vinyl stretching and bending modes are reported in orange and the ν(Fe‐Im) in red. The different irreducible representations of the other modes are shown with the following colors: A_1g_ (purple), A_2g_ (magenta), B_1g_ (maroon), and E_u_ (green). Arrows indicate the bands whose intensities increase or decrease upon addition of an excess of H_2_O_2_ [Colour figure can be viewed at wileyonlinelibrary.com]

Figure 7 shows the RR spectra of ferrous *Cd*ChdC coproheme (left) and heme *b* (right) complexes in presence of an excess of H_2_O_2_. In general, the effects on the spectra are very similar to those observed for the corresponding ferric forms. Moreover, because the complexes are 5cHS species, the ν(Fe‐Im) stretching mode is enhanced upon Soret excitation.^[^ ^43^ ^]^ Interestingly, its upshift from 210 to 215 cm^−1^, observed only in the heme *b* complex, suggests a slight strengthening of this bond when an excess of H_2_O_2_ is present. In contrast, the Y135A variant coproheme complex is characterized by a higher ν(Fe‐Im) wavenumber than the WT (215 vs. 203 cm^−1^), although their spectra display otherwise essentially identical features.^[^ ^44^ ^]^ This is probably due to a change in H‐bonding involving the propionate at position 2, which might affect the proximal coproheme side in the protein cavity lacking the Y135 residue that has its side chain pointed toward the β‐carbon of the propionate at position 2 in the WT.

## 4 DISCUSSION

The excess of hydrogen peroxide may influence either the heme porphyrin macrocycle or its peripheral substituents.

Structural changes that could affect the heme and possibly reduce its effective symmetry might derive from an out‐of‐plane distortion leading to a quasi S_4_ symmetry, as reported for saddle shape‐distorted cytochrome c^[^ ^41^, ^45^, ^46^ ^]^ or human myeloperoxidase.^[^ ^42^ ^]^ In this case, the A_2g_ bands, usually intensified only upon Q‐band excitation, become active also upon Soret excitation. Furthermore, some Raman bands can also exhibit a significant dispersion of their depolarization ratios upon symmetry lowering,^[^ ^45^, ^46^ ^]^ and, in addition, activation of porphyrin out‐of‐plane modes can be observed in the presence of out‐of‐plane distortions of the heme macrocycle.^[^ ^47^, ^48^ ^]^

Another structural change that could affect the heme is the removal of the fourfold rotation axis with conservation of a twofold rotational symmetry along one of the C_2_′ axes (C_2_(*x*) or C_2_(*y*)), as observed for iron chlorin formation.^[^ ^25^ ^]^ In this case, the A_2g_ representation of the D_4h_ pseudosymmetry group correlates to the B representations of the C_2_′ symmetry group, whereas the B_1g_ (D_4h_) representation correlates to the A (C_2_′) representation. Therefore, the A_2g_ modes, when Raman active, should correspond to depolarized bands and the B_1g_ modes should become polarized. By contrast, a reaction that affects the heme substituents would directly influence their Raman bending and stretching bands, without involving the heme skeletal vibrations.

The effect of the porphyrin distortions was investigated in great detail by Jentzen and coworkers using a combination of X‐ray crystallography, molecular mechanics calculations, and RR spectroscopy. They introduced a new normal structural decomposition method for classifying and quantifying the out‐of‐plane and in‐plane distortions. This method characterizes the distortion in terms of equivalent displacements along the normal coordinates of the D_4h_‐symmetric porphyrin macrocycle. Theoretically, the out‐of‐plane and in‐plane normal modes of each symmetry type form a basis for characterizing the (internal) displacements of the macrocyclic atoms.^[^ ^49^ ^]^

As mentioned above, many of the changes induced by the excess of hydrogen peroxide found in the RR spectra of the proteins under study are in common with those reported for cytochrome c spectra, except for the activation of out‐of‐plane skeletal modes (clearly not found in the present case). Therefore, the observed distortions must involve in‐plane normal deformations corresponding to the vibrational modes of the heme macrocycle, including the *x* and *y* pyrrole translations [*trn*(*x*), *trn*(*y*); E_u_] and pyrrole rotation (*rot*, A_2g_).^[^ ^49^ ^]^

We observe upon Soret excitation the appearance of a number of anomalously polarized bands for both the heme *b* and coproheme complexes in the presence of an excess of hydrogen peroxide. These A_2g_ bands also display a significant change in their polarization ratio. In addition, many E_u_ bands gain intensity and split into two components. Their activation and splitting derive from a heme distortion, which destroys the symmetry center. Further effects, confirming the presence of a twofold symmetry (not found in cytochrome c spectra), are the intensity decrease of the non‐totally symmetric modes B_1g_ (observed upon Q excitation) and the change of their polarization ratio (clearly observed for the isolated ν_10_ mode upon either Soret or Q‐band excitations).

Similar spectral changes were found by Andersson and coworkers for chlorins.^[^ ^25^ ^]^ In agreement with the reduction in the molecular symmetry from an effective D_4h_ for metalloporphyrins to an effective C_2_(*x*) for metallochlorins, the following changes should be expected: (i) the B_lg_ modes become polarized (i.e., as totally symmetric modes) in the chlorin spectra, (ii) the A_2g_ modes become B depolarized modes, and (iii) E_u_ (double degenerate) porphyrin modes should be split into A and B components. Of remarkable relevance is the finding of two intense “chlorin” bands present at lower and higher wavenumbers than that of the ν_4_ mode, as in our case.

On the basis of the similarities between the RR spectra of chlorin models and those observed in both systems under study, we can conclude that the excess hydrogen peroxide causes a porphyrin hydroxylation of ring C or D, which is compatible with the formation of an iron chlorin‐type heme *d* species. Reactions involving the vinyl groups (located on rings A and B) can be excluded because their corresponding modes are still evident in the RR spectra of the heme *b* complex after hydrogen peroxide treatment, although a slight readjustment is suggested by the wavenumber changes of both their stretching and bending modes. This is consistent also with both the electronic absorption spectra, which show a new band in the visible region but no change in the wavelength of the Soret band, and with the mass spectra, which indicate that the new species differ from the corresponding native porphyrin by the addition of an oxygen. Iron hydroxychlorins with saturation of a double bond on ring D have been proposed for metmyoglobin,^[^ ^13^ ^]^ whereas for catalase HPII^[^ ^50^ ^]^ from *E. coli* and bacterial terminal oxidase,^[^ ^20^ ^]^ iron hydroxychlorins on ring C have been isolated and characterized.

**FIGURE 8:**
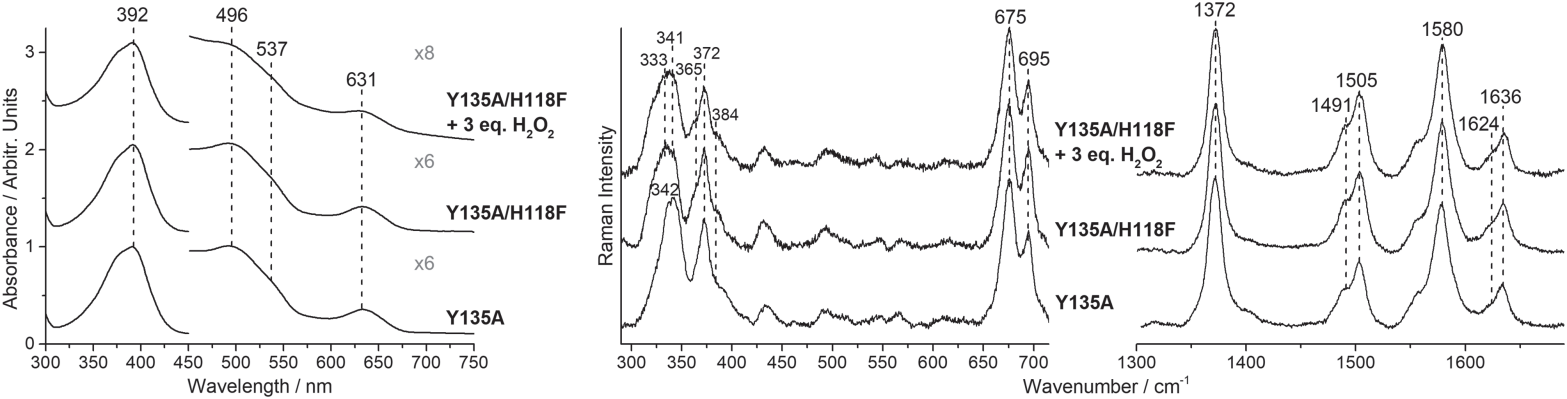
UV–vis (left) and resonance Raman spectra (center and right) of the ferric single Y135A and double Y135A/H118F variants of the *Cd*ChdC coproheme complex. The top spectra are obtained in the presence of an excess of hydrogen peroxide. All the UV–vis electronic absorption spectra have been normalized to the intensity of the Soret band, and the 450–700 nm region has been magnified as indicated

Interestingly, for catalase HPII, it has been reported that the distal His residue is an absolute requirement for hydroxylation of the protoheme.^[^ ^16^, ^18^ ^]^ The same conclusion can be drawn for the *Cd*ChdC, which uses a distal histidine residue (H118) as a base for deprotonation and subsequent heterolytic cleavage of hydrogen peroxide.^[^ ^23^ ^]^ In fact, in the absence of this distal His, an excess of hydrogen peroxide does not cause any spectroscopic changes. The single variants H118A and H118F, which are still catalytically active, are unaffected by an excess of hydrogen peroxide (data not shown) and do not show the oxidized porphyrin species. In addition, the catalytically inactive double variant Y135A/H118F coproheme complex gives rise to spectra identical to those of the single variant Y135A and the addition of hydrogen peroxide does not alter its spectral features (Figure 8). This is in agreement with previous observations, which showed that oxidation of the pyrrole ring is mediated by Compound I.^[^ ^13^ ^]^ In the absence of the distal Lewis base (H118), deprotonation of the incoming hydrogen peroxide is hampered and there is only negligible Compound I formation observable.^[^ ^23^ ^]^ In the *Cd*ChdC Y135A variant, the formation of the oxidized coproheme is only slightly slower (7.2 × 10^3^ M^−1^ s^−1^) (Figure 3, right) than Compound I formation (1.5 × 10^4^ M^−1^ s^−1^).^[^ ^23^ ^]^ Furthermore, in firmicute ChdC representatives, which do not have a residue on the distal side, which is capable of deprotonating hydrogen peroxide, no oxidation of coproheme nor heme *b* was observed.^[^ ^3^, ^4^ ^]^ WT *Cd*ChdC covalently links the oxidized form of heme *b* (630.5 Da; Figure 4b), whereas in *Lm*ChdC, heme *b* is cross‐linked without any modification (613.3 Da).^[^ ^51^ ^]^ Possibly, this cross‐linking of the heme is a rescue mechanism in vivo, if for some reason hydrogen peroxide concentrations rise to an undesirable level. In this case, the cross‐linking would stop the heme biosynthesis of the respective organism because the cross‐linked heme cannot be released into the cytoplasm. Free heme in the cell is of course a threat and can cause unwanted oxidative side reactions.^[^ ^52^ ^]^ The fact that in actinobacterial *Cd*ChdC the oxidized species is formed prior to cross‐linking only reflects the overall higher reactivity toward hydrogen peroxide, compared with the so far investigated firmicute ChdCs.

## 5 CONCLUSIONS

This work highlights the capability of RR spectroscopy to identify the presence of H_2_O_2_‐induced side reactions on heme proteins, which may alter their structure and functionality. Its sensitivity allowed us to correlate the observed intensity and polarization ratio changes of various vibrational modes to the symmetry lowering of the porphyrin ring from pseudosymmetry D_4h_ to C_2_′, which causes a substantial distortion of the porphyrin ring, involving in‐plane normal deformations. The vibrational data together with electronic absorption and MS indicate that for the coproheme and heme *b* complexes of the *Cd*ChdC, an excess of hydrogen peroxide causes a porphyrin hydroxylation of ring C or D. This important result is fully compatible with the formation of iron chlorin‐type heme *d* species and correlates with a potential protection mechanism from oxidative damage. A similar effect has been previously observed for other heme‐containing proteins, but this is the first time that a similar mechanism is reported for a coproheme enzyme. The hydroxylation needs the presence of the distal histidine residue (H118), which is also important for the oxidative decarboxylation because this residue acts as a base for deprotonation and subsequent heterolytic cleavage of hydrogen peroxide.

## Supporting information




**Figure S1.** Comparison of the UV–vis electronic absorption (top panel) and RR
spectra in the low and high wavenumber regions (bottom panels) of the ferric *Cd*ChdC coproheme‐complexes of wild‐type (WT) and Y135A variant. The UV–vis electronic absorption spectra have been normalized to the maximum intensity of the Soret band and the 450–700 nm region has been magnified as indicated in grey.
**Table S1.** Integration time and number of averaged spectra reported in the figures for the WT *Cd*ChdC and investigated variant complexes in the ferric and ferrous forms. The light polarization is reported only (par. for parallel and per. for perpendicular), when different from non‐polarized.

## Data Availability

No data ara available.
