# Supplementary material for: Spectroscopic evidence of the effect of hydrogen peroxide excess on the coproheme decarboxylase from actinobacterial Corynebacterium diphtheriae
Source: J Raman Spectrosc. 2022 Mar 8;53(5):890–901. doi: 10.1002/jrs.6326 (PMC9310987; doi:10.1002/jrs.6326)
Supplement: Supplementary file 1 — Figure S1. Comparison of the UV–vis electronic absorption (top panel) and RR spectra in the low and high wavenumber regions (bottom panels) of the ferric CdChdC coproheme‐complexes of wild‐type (WT) and Y135A variant. The UV–vis electronic absorption spectra have been normalized to the maximum intensity of the Soret band and the 450–700 nm region has been magnified as indicated in grey. Table S1. Integration time and number of averaged spectra reported in the figures for the WT CdChdC and investigated variant complexes in the ferric and ferrous forms. The light polarization is reported only (par. for parallel and per. for perpendicular), when different from non‐polarized. [file JRS-53-890-s001.docx]

**Spectroscopic evidence of the effect of hydrogen peroxide excess on the coproheme decarboxylase from actinobacterial *Corynebacterium diphtheriae***

Federico Sebastiani^1^, Chiara Niccoli^1^, Hanna Michlits^2^, Riccardo Risorti^1^, Maurizio Becucci^1*^, Stefan Hofbauer^2^, and Giulietta Smulevich^1,3^*

^1^Dipartimento di Chimica “Ugo Schiff” DICUS, Università di Firenze, Via della Lastruccia 3-13, I-50019 Sesto Fiorentino (FI), Italy

^2^University of Natural Resources and Life Sciences, Vienna, Department of Chemistry, Institute of Biochemistry, Muthgasse 18, A-1190 Vienna, Austria

^3^INSTM Research Unit of Firenze, via della Lastruccia 3, I-50019 Sesto Fiorentino, Italy

Corresponding Authors

* e-mail: giulietta.smulevich@unifi.it, phone: +39 055 4573083, ORCID: 0000-0003-3021-8919

e-mail: [maurizio.becucci@unifi.it](mailto:maurizio.becucci@unifi.it), phone: +39 055 4573089, ORCID: **0000-0002-2428-471X**

**SUPPLEMENTAL MATERIALS**

**Figure S1.** Comparison of the UV-vis electronic absorption (top panel) and RR
spectra in the low and high wavenumber regions (bottom panels) of the ferric *Cd*ChdC coproheme-complexes of wild-type (WT) and Y135A variant. The UV-vis electronic absorption spectra have been normalized to the maximum intensity of the Soret band and the 450-700 nm region has been magnified as indicated in grey.

**

**

**Table S1.** Integration time and number of averaged spectra reported in the figures for the WT *Cd*ChdC and investigated variant complexes in the ferric and ferrous forms. The light polarization is reported only (par. for parallel and per. for perpendicular), when different from non-polarized.

| **Protein** | **Oxidation**  **State** |  | **λ_exc_** | **Low Wavenumber**  **(average/**  **integration time)** | **High Wavenumber**  **(average/**  **integration time)** |
| --- | --- | --- | --- | --- | --- |
| **WT** | Fe(III) | coproheme | 406.7 nm  532 nm | 50 spectra/250 min | 36 spectra/180 min  20 spectra/100 min (par)  22 spectra/110 min (per)  24 spectra/60 min  12 spectra/60 min (par)  12 spectra/60 min (per) |
|  | Fe(III) | heme *b* | 406.7 nm  532 nm | 24 spectra/120 min | 24 spectra/120 min  24 spectra/120 min (par)  24 spectra/120 min (per)  80 spectra/160 min  15 spectra/75 min (par)  20 spectra/100 min (per) |
|  | Fe(III) | +3.5 eq. H_2_O_2_ | 406.7 nm  532 nm | 24 spectra/120 min | 24 spectra/120 min  20 spectra/100 min (par)  24 spectra/120 min (per)  100 spectra/250 min  110 spectra/220 min (par)  85 spectra/170 min (per) |
|  | Fe(II) | heme *b* | 441.6 nm | 24 spectra/48 min | 24 spectra/48 min |
|  | Fe(II) | +3.5 eq. H_2_O_2_ | 441.6 nm | 150 spectra/100 min | 150 spectra/100 min |
| **Y135A** | Fe(III) | coproheme | 406.7 nm | 28 spectra/140 min | 16 spectra/80 min |
|  | Fe(III) | +2 eq. H_2_O_2_ | 406.7 nm  532 nm | 28 spectra/140 min | 20 spectra/100 min  20 spectra/100 min (par)  20 spectra/100 min (per)  60 spectra/90 min  20 spectra/70 min (par)  20 spectra/70 min (per) |
|  | Fe(II) | coproheme | 441.6 nm  413.1 nm | 40 spectra/80 min  40 spectra/80 min | 40 spectra/80 min  40 spectra/80 min |
|  | Fe(II) | +2 eq. H_2_O_2_ | 413.1 nm | 50 spectra/100 min | 50 spectra/100 min |
| **Y135A/**  **H118F** | Fe(III) | coproheme | 406.7 nm | 32 spectra/160 min | 20 spectra/100 min |
|  | Fe(III) | +3 eq. H_2_O_2_ | 406.7 nm | 32 spectra/160 min | 16 spectra/80 min |
